# Supplementary material for: Hybrid Models and Biological Model Reduction with PyDSTool
Source: PLoS Comput Biol. 2012 Aug 9;8(8):e1002628. doi: 10.1371/journal.pcbi.1002628 (PMC3415397; doi:10.1371/journal.pcbi.1002628)
Supplement: Text S4 — Complete source code for the PyDSTool package (version 0.88.120504). Includes API documentation and help files linking to web pages. This file is identical to the current public release on Sourceforge.net. (ZIP) [file pcbi.1002628.s004.zip › PyDSTool/html/PyDSTool.Toolbox.neuro_data-pysrc.html]

xml version="1.0" encoding="ascii"?


PyDSTool.Toolbox.neuro\_data


| Home | Trees | Indices | Help | PyDSTool |
| --- | --- | --- | --- | --- |

Package PyDSTool ::
Package Toolbox ::
Module neuro\_data

[hide private]

[frames] | no frames]

# Source Code for Module PyDSTool.Toolbox.neuro\_data

```
   1  import numpy as npy 
   2  from PyDSTool import Events, plot, show, figure, Variable, Pointset, Trajectory 
   3  from PyDSTool.common import args, metric, metric_L2, metric_weighted_L2, \ 
   4       metric_float, remain, fit_quadratic, fit_exponential, fit_diff_of_exp, \ 
   5       smooth_pts, nearest_2n_indices, make_poly_interpolated_curve, simple_bisection 
   6  from PyDSTool.Trajectory import numeric_to_traj 
   7  from PyDSTool.MProject import * 
   8  from PyDSTool.Toolbox.data_analysis import butter, filtfilt, rectify 
   9  from PyDSTool.errors import PyDSTool_KeyError 
  10  import copy 
  11   
  12  # Test this on a single spike with global max at spike and minima at endpoints 
  13  # Test this on a mexican hat type spike with global min and max at spike peak and trough 
  14  # Test this on monotonic data for worst case scenario!! Should return None for max and min 
  15  # Also test on noisy monotonic data 
  16  # Return value of Nones to a feature evaluator should suggest to it to change window size for defining pts 


17 -def find_internal_extrema(pts, noise_tol=0):


18      """ 
  19      Find an interior (local) maximum and minimum values of a 1D pointset, away from the endpoints. 
  20      Returns a dictionary mapping 'local_max' -> (index_max, xmax), 'local_min' -> (index_min, xmin), 
  21      whose values are None if the pointset is monotonic or is close enough so that the global extrema 
  22      are at the endpoints. 
  23   
  24      Use noise_tol > 0 to avoid getting a local extremum right next to an endpoint because of noise. 
  25   
  26      Also returned in the dictionary for reference: 
  27      'first' -> (0, <start_endpoint_value>), 'last' -> (last_index, <last_endpoint_value>), 
  28      'global_max' -> (index, value), 'global_min' -> (index, value) 
  29   
  30      Assumes there is only one interior (max, min) pair in pts, otherwise will return an arbitrary choice 
  31      from multiple maxima and minima.""" 
  32   
  33      assert pts.dimension == 1 
  34      # convert all singleton points to floats with [0] selection 
  35      x0 = pts[0][0] 
  36      x1 = pts[-1][0] 
  37      # need last_ix explicitly for index test below 
  38      last_ix = len(pts)-1 
  39      end_ixs = (0, last_ix) 
  40   
  41      max_val_ix = npy.argmax(pts) 
  42      min_val_ix = npy.argmin(pts) 
  43      glob_xmax = pts[max_val_ix][0] 
  44      glob_xmin = pts[min_val_ix][0] 
  45   
  46      no_local_extrema = {'local_max': (None, None), 'local_min': (None, None), 
  47                          'first': (0, x0), 'last': (last_ix, x1), 
  48                          'global_max': (max_val_ix, glob_xmax), 
  49                          'global_min': (min_val_ix, glob_xmin) 
  50                      } 
  51   
  52      max_at_end = max_val_ix in end_ixs 
  53      min_at_end = min_val_ix in end_ixs 
  54      if max_at_end: 
  55          if min_at_end: 
  56              # No detectable turning points present (this is criterion for ignoring noisy data) 
  57              return no_local_extrema 
  58          else: 
  59              # interior minimum found 
  60              index_min = min_val_ix 
  61              xmin = pts[index_min] 
  62              # find associated interior local maximum 
  63              max_val_ix1 = npy.argmax(pts[:min_val_ix]) 
  64              max_val_ix2 = npy.argmax(pts[min_val_ix:])+min_val_ix 
  65              if max_val_ix1 in end_ixs: 
  66                  if max_val_ix2 in end_ixs: 
  67                      index_max = None 
  68                      xmax = None 
  69                  else: 
  70                      index_max = max_val_ix2 
  71                      xmax = pts[index_max][0] 
  72              else: 
  73                  # assumes only one local max / min pair in interior! 
  74                  index_max = max_val_ix1 
  75                  xmax = pts[index_max][0] 
  76      else: 
  77          # interior maximum found 
  78          index_max = max_val_ix 
  79          xmax = pts[index_max][0] 
  80          # find associated interior local minimum 
  81          min_val_ix1 = npy.argmin(pts[:max_val_ix]) 
  82          xmin1 = pts[min_val_ix1][0] 
  83          min_val_ix2 = npy.argmin(pts[max_val_ix:])+max_val_ix 
  84          xmin2 = pts[min_val_ix2][0] 
  85          if min_val_ix1 in end_ixs or abs(xmin1-x0)<noise_tol or abs(xmin1-x1)<noise_tol: 
  86              if min_val_ix2 in end_ixs or abs(xmin1-x0)<noise_tol or abs(xmin1-x1)<noise_tol: 
  87                  index_min = None 
  88                  xmin = None 
  89              else: 
  90                  index_min = min_val_ix2 
  91                  xmin = xmin2 
  92          else: 
  93              # assumes only one local max / min pair in interior! 
  94              index_min = min_val_ix1 
  95              xmin = xmin1 
  96   
  97      return {'local_max': (index_max, xmax), 'local_min': (index_min, xmin), 
  98              'first': (0, x0), 'last': (last_ix, x1), 
  99              'global_max': (max_val_ix, glob_xmax), 
 100              'global_min': (min_val_ix, glob_xmin)}

 101   
 102   


103 -class get_spike_model(ql_feature_leaf):


104      """Qualitative test for presence of spike in model trajectory data 
 105       using events to identify spike times. Also records salient spike 
 106       information for quantitative comparisons later.""" 
 107   


108 -    def evaluate(self, traj):


109          # function of traj, not target 
 110          pts = traj.sample(coords=[self.super_pars.burst_coord], 
 111                                        tlo=self.pars.tlo, 
 112                            thi=self.pars.tlo+self.pars.width_tol) 
 113          loc_extrema = find_internal_extrema(pts) 
 114          if self.pars.verbose_level > 0: 
 115              print loc_extrema 
 116          max_val_ix, xmax = loc_extrema['local_max'] 
 117          global_max_val_ix, global_xmax = loc_extrema['global_max'] 
 118          min_val_ix, xmin = loc_extrema['local_min'] 
 119          global_min_val_ix, global_xmin = loc_extrema['global_min'] 
 120   
 121          # could split these tests into 3 further sub-features but we'll skip that here for efficiency 
 122          if xmax is None: 
 123              self.results.ixmax = None 
 124              self.results.tmax = None 
 125              test1 = test2 = test3 = False 
 126          else: 
 127              test1 = max_val_ix not in (loc_extrema['first'][0], loc_extrema['last'][0]) 
 128              test2 = npy.linalg.norm(global_xmin-xmax) > self.pars.height_tol 
 129              try: 
 130                  test3 = npy.linalg.norm(xmin-xmax) > self.pars.height_tol 
 131              except: 
 132                  # fails if xmin is None, i.e. no interior minimum 
 133                  # allow no local minimum present, in which case use the other endpoint for test 
 134                  # ... we don't know which is the one alread tested in test2, so test both ends again, 
 135                  # knowing that they are both lower than the interior maximum found in this case 
 136                  xmin = max([global_xmin, loc_extrema['last'][1], loc_extrema['first'][1]]) 
 137                  test3 = npy.linalg.norm(xmin-xmax) > self.pars.height_tol 
 138              self.results.ixmax = max_val_ix 
 139              self.results.tmax = pts.indepvararray[max_val_ix] 
 140          self.results.spike_pts = pts 
 141          return test1 and test2 and test3

 142   


143 -    def finish(self, traj):


144          self.results.spike_time = self.results.tmax 
 145          self.results.spike_val = self.results.spike_pts[self.results.ixmax][self.super_pars.burst_coord]

 146   
 147   


148 -class get_spike_data(ql_feature_leaf):


149      """Qualitative test for presence of spike in noisy data. Also records salient spike information 
 150       for quantitative comparisons later. 
 151   
 152      Criteria: ensure a maximum occurs, and that this is away from endpoints of traj 
 153       "Uniqueness" of this maximum can only be determined for noisy data using a height 
 154       tolerance. 
 155   
 156      Assumes spikes will never bunch up too much so that more than spike occurs in the 
 157       spacing_tol window. 
 158   
 159      Finds maximum position using a quadratic fit. 
 160      """ 


161 -    def _local_init(self):


162          # avoids recreating this object for every test 
 163          self.quadratic = fit_quadratic(verbose=self.pars.verbose_level>0)

 164   


165 -    def evaluate(self, traj):


166          # function of traj, not target 
 167          event_args = {'name': 'spike_thresh', 
 168                 'eventtol': self.pars.eventtol, 
 169                 'eventdelay': self.pars.eventtol*.1, 
 170                 'starttime': 0, 
 171                 'active': True} 
 172          if 'coord' not in self.pars: 
 173              self.pars.coord = self.super_pars.burst_coord 
 174          # update thi each time b/c tlo will be different 
 175          self.pars.thi = self.pars.tlo+self.pars.width_tol 
 176          self.pars.ev = Events.makePythonStateZeroCrossEvent(self.pars.coord, 
 177                                                              "thresh", 0, 
 178                                      event_args, traj.variables[self.pars.coord]) 
 179          pts = traj.sample(coords=[self.pars.coord], tlo=self.pars.tlo, 
 180                                thi=self.pars.thi) 
 181          if pts.indepvararray[-1] < self.pars.thi: 
 182              self.pars.thi = pts.indepvararray[-1] 
 183          loc_extrema = find_internal_extrema(pts, self.pars.noise_tol) 
 184          if self.pars.verbose_level > 0: 
 185              print loc_extrema 
 186              ## plot spike and quadratic fit 
 187              #plot(pts.indepvararray, pts[self.super_pars.burst_coord], 'go-') 
 188              #show() 
 189          max_val_ix, xmax = loc_extrema['local_max'] 
 190          global_max_val_ix, global_xmax = loc_extrema['global_max'] 
 191          min_val_ix, xmin = loc_extrema['local_min'] 
 192          global_min_val_ix, global_xmin = loc_extrema['global_min'] 
 193   
 194          # could split these tests into 3 further sub-features but we'll skip that here for efficiency 
 195          test1 = max_val_ix not in (loc_extrema['first'][0], loc_extrema['last'][0]) 
 196          test2 = npy.linalg.norm(global_xmin-xmax) > self.pars.height_tol 
 197          try: 
 198              test3 = npy.linalg.norm(xmin-xmax) > self.pars.height_tol 
 199          except: 
 200              # fails if xmin is None, i.e. no interior minimum 
 201              # allow no local minimum present, in which case use the other endpoint for test 
 202              # ... we don't know which is the one already tested in test2, so test both ends again, 
 203              # knowing that they are both lower than the interior maximum found in this case 
 204              xmin = max([global_xmin, loc_extrema['last'][1], loc_extrema['first'][1]]) 
 205              test3 = npy.linalg.norm(xmin-xmax) > self.pars.height_tol 
 206          # generate a suitable threshold from local maximum 
 207          try: 
 208              thresh_pc = self.pars.thresh_pc 
 209          except: 
 210              # default value of 15% 
 211              thresh_pc = 0.15 
 212          thresh = (xmin + thresh_pc*(xmax-xmin)) 
 213          if self.pars.verbose_level > 0: 
 214              print "xmin used =", xmin 
 215              print "thresh = ", thresh 
 216          # Define extent of spike for purposes of quadratic fit ... 
 217          evs_found = self.pars.ev.searchForEvents(trange=[self.pars.tlo, 
 218                                                           self.pars.thi], 
 219                                         parDict={'thresh': thresh}) 
 220          tlo = evs_found[0][0] 
 221          thi = evs_found[1][0] 
 222          tmax = pts.indepvararray[max_val_ix] 
 223          symm_dist = npy.min([abs(tmax-tlo), abs(thi-tmax)]) 
 224          # HACK! Ensure dt value will not cause us to hit an index directly, otherwise 
 225          # have to catch case from Pointset.find method when return value is a single 
 226          # integer index rather than a pair of indices 
 227          if symm_dist > self.pars.fit_width_max/2.000000007: 
 228              dt = self.pars.fit_width_max/2.000000007 
 229          else: 
 230              dt = symm_dist*1.0000000007 
 231          tlo = tmax-dt 
 232          thi = tmax+dt 
 233          ixlo = pts.find(tmax-dt, end=0) 
 234          ixhi = pts.find(tmax+dt, end=1) 
 235          if self.pars.verbose_level > 0: 
 236              print "ixlo =", ixlo, "ixhi =", ixhi 
 237              print "tlo =",tmax-dt, "thi =",tmax+dt 
 238              print pts[ixlo], pts[ixhi] 
 239              print "\nget_spike tests:", test1, test2, test3 
 240          self.results.ixlo = ixlo 
 241          self.results.ixhi = ixhi 
 242          self.results.ixmax = max_val_ix 
 243          self.results.tlo = tlo 
 244          self.results.thi = thi 
 245          self.results.tmax = tmax 
 246          self.results.spike_pts = pts[ixlo:ixhi] 
 247          return test1 and test2 and test3

 248   


249 -    def finish(self, traj):


250          # function of traj, not target 
 251          if self.pars.verbose_level > 0: 
 252              print "Finishing spike processing..." 
 253          pts = self.results.spike_pts 
 254          coord = self.pars.coord 
 255          xlo = pts[0][0] 
 256          # xmax is just an estimate of the max value 
 257          xmax = pts[self.results.ixmax-self.results.ixlo][0] 
 258          estimate_quad_coeff = -(xmax-xlo)/((self.results.tmax - \ 
 259                                              self.results.tlo)**2) 
 260          estimate_intercept = xlo - \ 
 261                ((xmax-xlo)/(self.results.tmax-self.results.tlo))*self.results.tlo 
 262          res = self.quadratic.fit(pts.indepvararray, pts[coord], 
 263                              pars_ic=(estimate_quad_coeff,0,estimate_intercept), 
 264                              opts=args(peak_constraint=(self.results.ixmax - \ 
 265                                                         self.results.ixlo,xmax, 
 266                                  self.pars.weight*len(pts)/(self.results.tmax - \ 
 267                                                             self.results.tlo), 
 268                                  self.pars.weight*len(pts)/(xmax-xlo)))) 
 269          tval, xval = res.results.peak 
 270          self.results.spike_time = tval 
 271          self.results.spike_val = xval 
 272          self.results.pars_fit = res.pars_fit 
 273          if self.pars.verbose_level > 0: 
 274              # plot spike and quadratic fit 
 275              dec = 10 
 276              plot(pts.indepvararray, pts[coord], 'go-') 
 277              plot(tval, xval, 'rx') 
 278              ts = [pts.indepvararray[0]] 
 279              for i, t in enumerate(pts.indepvararray[:-1]): 
 280                  ts.extend([t+j*(pts.indepvararray[i+1]-t)/dec for j in range(1,dec)]) 
 281              ts.append(pts.indepvararray[-1]) 
 282              plot(ts, [res.results.f(t) for t in ts], 'k:') 
 283              # temp 
 284              if self.pars.verbose_level > 1: 
 285                  show()

 286   
 287   
 288   


289 -class get_burst_duration(qt_feature_leaf):


290 -    def _local_init(self):


291          self.metric = metric_float() 
 292          self.metric_len = 1

 293   


294 -    def postprocess_ref_traj(self):


295          on_t = self.super_pars.ref_spike_times[0] - self.pars.t_lookback 
 296          self.pars.ref_burst_on_time = on_t 
 297          # find associated V for ref_on_thresh 
 298          pts = self.super_pars.ref_burst_coord_pts 
 299          x = pts[self.super_pars.burst_coord] 
 300          on_ix = pts.find(on_t, end=1) 
 301          ix_lo, ix_hi = nearest_2n_indices(x, on_ix, 2) 
 302          t = pts.indepvararray 
 303          on_res = smooth_pts(t[ix_lo:ix_hi+1], 
 304                              x[ix_lo:ix_hi+1], self.super_pars.quadratic) 
 305          self.pars.ref_on_thresh = on_res.results.f(on_t) 
 306          # 
 307          off_t = self.super_pars.ref_spike_times[-1] + self.pars.t_lookforward 
 308          self.pars.ref_burst_off_time = off_t 
 309          off_ix = pts.find(off_t, end=0) 
 310          ix_lo, ix_hi = nearest_2n_indices(x, off_ix, 2) 
 311          off_res = smooth_pts(t[ix_lo:ix_hi+1], 
 312                               x[ix_lo:ix_hi+1], self.super_pars.quadratic) 
 313          self.pars.ref_off_thresh = off_res.results.f(off_t) 
 314          self.pars.ref_burst_duration = off_t - on_t 
 315          self.pars.ref_burst_prop = (off_t - on_t)/self.super_pars.ref_period

 316   


317 -    def evaluate(self, target):


318          traj = target.test_traj 
 319          varname = self.super_pars.burst_coord 
 320          pts = self.super_pars.burst_coord_pts 
 321          on_t = self.super_results.spike_times[0] - self.pars.t_lookback 
 322          self.results.burst_on_time = on_t 
 323          x = pts[varname] 
 324          on_ix = pts.find(on_t, end=1) 
 325          ix_lo, ix_hi = nearest_2n_indices(x, on_ix, 2) 
 326          pp = make_poly_interpolated_curve(pts[ix_lo:ix_hi+1], varname, 
 327                                            target.model) 
 328          thresh = pp(on_t) 
 329          self.results.on_thresh = thresh 
 330          # 
 331          # don't find "off" based on last spike time because 
 332          # when new spikes suddenly appear this value will jump 
 333          # instead, use a threshold event search, assuming that 
 334          # only one period is "in view" 
 335          t = pts.indepvararray 
 336          x_rev = x[:ix_hi:-1] 
 337          t_rev = t[:ix_hi:-1] 
 338          off_ix = len(x) - npy.argmin(npy.asarray(x_rev < thresh, int)) 
 339          ix_lo, ix_hi = nearest_2n_indices(x, off_ix, 2) 
 340          pp = make_poly_interpolated_curve(pts[ix_lo:ix_hi+1], varname, 
 341                                            target.model) 
 342          # bisect to find accurate crossing point 
 343          tlo = t[ix_lo] 
 344          thi = t[ix_hi] 
 345          off_t = simple_bisection(tlo, thi, pp, self.pars.t_tol) 
 346          self.results.burst_duration = off_t - on_t 
 347          self.results.burst_prop = (off_t - on_t) / self.super_results.period 
 348          return self.metric(self.results.burst_prop, 
 349                             self.super_pars.ref_burst_prop) < self.pars.tol

 350   
 351   


352 -class get_burst_active_phase(qt_feature_leaf):


353 -    def _local_init(self):


354          self.metric = metric_float() 
 355          self.metric_len = 1

 356   


357 -    def postprocess_ref_traj(self):


358          self.pars.ref_active_phase = self.super_pars.ref_spike_times[0] / \ 
 359              self.super_pars.ref_period

 360   


361 -    def evaluate(self, target):


362          self.results.active_phase = self.super_results.spike_times[0] / \ 
 363              self.super_results.period 
 364          return self.metric(self.results.active_phase, 
 365                             self.pars.ref_active_phase) \ 
 366                 < self.pars.tol

 367   


368 -class get_burst_dc_offset(qt_feature_leaf):


369 -    def _local_init(self):


370          self.metric = metric_float() 
 371          self.metric_len = 1

 372   


373 -    def postprocess_ref_traj(self):


374          # 20% of burst_on_V (i.e., on_thresh) - min_V above min_V 
 375          self.pars.ref_baseline_V = self.super_pars.ref_min_V + \ 
 376                                 0.2*(self.super_pars.ref_on_thresh - \ 
 377                                      self.super_pars.ref_min_V)


378 -    def evaluate(self, target):


379          baseline = self.super_results.min_V + 0.2*(self.super_results.on_thresh - \ 
 380                                                  self.super_results.min_V) 
 381          self.results.baseline_V = baseline - self.super_pars.ref_baseline_V 
 382          return self.metric(baseline, self.super_pars.ref_baseline_V) < \ 
 383                                 self.pars.tol

 384   
 385   


386 -class get_burst_passive_extent(qt_feature_leaf):


387 -    def _local_init(self):


388          self.metric = metric_float() 
 389          self.metric_len = 1

 390   


391 -    def postprocess_ref_traj(self):


392          self.pars.ref_passive_extent_V = self.super_pars.ref_max_V - \ 
 393                     self.super_pars.ref_min_V

 394   


395 -    def evaluate(self, target):


396          self.results.passive_extent_V = self.super_results.max_V - \ 
 397              self.super_results.min_V 
 398          return self.metric(self.results.passive_extent_V, 
 399                             self.super_pars.ref_passive_extent_V) < \ 
 400                                 self.pars.tol

 401   
 402   


403 -class burst_feature(ql_feature_node):


404      """Embed the following sub-features, if desired: 
 405      get_burst_X, where X is a number of feature types defined in this module. 
 406      """ 


407 -    def _local_init(self):


408          self.pars.quadratic = fit_quadratic(verbose=self.pars.verbose_level>0) 
 409          self.pars.filt_coeffs = butter(3, self.pars.cutoff, btype='highpass') 
 410          self.pars.filt_coeffs_LP = butter(3, self.pars.cutoff/10)

 411   


412 -    def postprocess_ref_traj(self):


413          # single coord used as indicator 
 414          pts = self.ref_traj.sample() 
 415          burst_pts = self.ref_traj.sample(coords=[self.pars.burst_coord], 
 416                              dt=self.pars.dt) 
 417          xrs = burst_pts[self.pars.burst_coord] 
 418          trs = burst_pts.indepvararray 
 419          x = pts[self.pars.burst_coord] 
 420          b, a = self.pars.filt_coeffs_LP 
 421          xf = filtfilt(b, a, xrs) 
 422          t = pts.indepvararray 
 423          min_val_ix = npy.argmin(xf)  # use LPF version to avoid noise artifacts 
 424          max_val_ix = npy.argmax(xf)  # use LPF version to avoid spikes 
 425          min_ix_lo, min_ix_hi = nearest_2n_indices(xrs, min_val_ix, 30) 
 426          max_ix_lo, max_ix_hi = nearest_2n_indices(xrs, max_val_ix, 30) 
 427          min_res = smooth_pts(trs[min_ix_lo:min_ix_hi+1], 
 428                               xf[min_ix_lo:min_ix_hi+1], self.pars.quadratic) 
 429          # use LPF data for max 
 430          max_res = smooth_pts(trs[max_ix_lo:max_ix_hi+1], 
 431                               xf[max_ix_lo:max_ix_hi+1], self.pars.quadratic) 
 432          min_t, min_val = min_res.results.peak 
 433          max_t, max_val = max_res.results.peak 
 434  #        thresh1 = float(max_val-self.pars.active_frac_height*(max_val-min_val)) 
 435  #        thresh2 = x[0]+3. 
 436  #        # don't make threshold smaller than initial value, assuming 
 437  #        # burst will be rising at initial condition 
 438  #        thresh = max((thresh1,thresh2)) 
 439          self.pars.ref_burst_coord_pts = pts 
 440  #        self.pars.ref_on_thresh = thresh 
 441  #        self.pars.ref_off_thresh = thresh 
 442          self.pars.ref_min_V = min_val 
 443          self.pars.ref_max_V = max_val 
 444          assert self.pars.on_cross_dir in (-1,1) 
 445          if self.pars.on_cross_dir == 1: 
 446              self.pars.off_cross_dir = -1 
 447          else: 
 448              self.pars.off_cross_dir = 1 
 449          self.pars.ref_burst_est = estimate_spiking(burst_pts[self.pars.burst_coord], 
 450                                       burst_pts.indepvararray, 
 451                                       self.pars.filt_coeffs) 
 452          self.pars.ref_burst_pts_resampled = burst_pts 
 453          # spike times will be overwritten by get_spikes_data instance, if present 
 454          #self.pars.ref_spike_times = self.pars.ref_burst_est.spike_ts 
 455                  # to establish period, find min on other side of active phase 
 456          if min_t < self.pars.ref_burst_est.spike_ts[0]: 
 457              # look to the right 
 458              start_t = self.pars.ref_burst_est.spike_ts[-1] 
 459              start_ix = pts.find(start_t, end=1) 
 460              other_min_ix = npy.argmin(x[start_ix:]) 
 461              other_min_t = t[start_ix+other_min_ix] 
 462          else: 
 463              # look to the left 
 464              start_t = self.pars.ref_burst_est.spike_ts[0] 
 465              start_ix = pts.find(start_t, end=0) 
 466              other_min_ix = npy.argmin(x[:start_ix]) 
 467              other_min_t = t[other_min_ix] 
 468          self.pars.ref_period = abs(other_min_t - min_t)

 469   
 470   


471 -    def prepare(self, target):


472          # single coord used as indicator 
 473          pts = target.test_traj.sample() 
 474          x = pts[self.pars.burst_coord] 
 475          burst_pts = target.test_traj.sample(coords=[self.pars.burst_coord], 
 476                              dt=self.pars.dt) 
 477          xrs = burst_pts[self.pars.burst_coord] 
 478          trs = burst_pts.indepvararray 
 479          if max(x)-min(x) < 5: 
 480              print "\n\n  Not a bursting trajectory!!" 
 481              raise ValueError("Not a bursting trajectory") 
 482          b, a = self.pars.filt_coeffs_LP 
 483          xf = filtfilt(b, a, xrs) 
 484          t = pts.indepvararray 
 485          min_val_ix = npy.argmin(x) # precise because of Model's events 
 486          max_val_ix = npy.argmax(xf) 
 487          max_ix_lo, max_ix_hi = nearest_2n_indices(xrs, max_val_ix, 4) 
 488          max_res = smooth_pts(trs[max_ix_lo:max_ix_hi+1], 
 489                               xf[max_ix_lo:max_ix_hi+1], self.pars.quadratic) 
 490          min_t = t[min_val_ix] 
 491          min_val = x[min_val_ix] 
 492          max_t, max_val = max_res.results.peak 
 493          self.results.min_V = min_val 
 494          self.results.max_V = max_val 
 495          assert self.pars.on_cross_dir in (-1,1) 
 496          if self.pars.on_cross_dir == 1: 
 497              self.pars.off_cross_dir = -1 
 498          else: 
 499              self.pars.off_cross_dir = 1 
 500          self.results.burst_est = estimate_spiking(burst_pts[self.pars.burst_coord], 
 501                                       burst_pts.indepvararray, 
 502                                       self.pars.filt_coeffs) 
 503          # record approximate spike times - may be overwritten by 
 504          # get_burst_spikes if done accurately 
 505          #self.results.spike_times = self.results.burst_est.spike_ts 
 506          if self.pars.verbose_level > 0: 
 507              print "Spikes found at (approx) t=", self.results.burst_est.spike_ts 
 508          if self.results.burst_est.spike_ts[0] < self.pars.shrink_end_time_thresh: 
 509              # kludgy way to ensure that another burst doesn't encroach 
 510              if not hasattr(self.pars, 'shrunk'): 
 511                  # do this *one time* 
 512                  end_time = t[-1] - self.pars.shrink_end_time_amount 
 513                  target.model.set(tdata=[0,end_time]) 
 514                  end_pts = pts.find(end_time, end=0) 
 515                  end_burst_pts = burst_pts.find(end_time, end=0) 
 516                  pts = pts[:end_pts] 
 517                  burst_pts = burst_pts[:end_burst_pts] 
 518                  self.pars.shrunk = True 
 519          elif hasattr(self.pars, 'shrunk'): 
 520              # in case period grows back reset end time *one time* 
 521              target.model.set(tdata=[0,t[-1]+self.pars.shrink_end_time_amount]) 
 522              del self.pars.shrunk 
 523          self.pars.burst_coord_pts = pts 
 524          self.pars.burst_pts_resampled = burst_pts 
 525          # to establish period, find min on other side of active phase 
 526          if min_t < self.results.burst_est.spike_ts[0]: 
 527              # look to the right 
 528              start_t = self.results.burst_est.spike_ts[-1] 
 529              start_ix = pts.find(start_t, end=1) 
 530              other_min_ix = npy.argmin(x[start_ix:]) 
 531              other_min_t = t[start_ix+other_min_ix] 
 532              other_min_val = x[start_ix+other_min_ix] 
 533          else: 
 534              # look to the left 
 535              start_t = self.results.burst_est.spike_ts[0] 
 536              start_ix = pts.find(start_t, end=0) 
 537              other_min_ix = npy.argmin(x[:start_ix]) 
 538              other_min_t = t[other_min_ix] 
 539              other_min_val = x[other_min_ix] 
 540          self.results.period = abs(other_min_t - min_t) 
 541          self.results.period_val_error = other_min_val - min_val

 542   
 543   


544 -class get_burst_spikes(ql_feature_node):


545      """Requires a get_spike_data and get_spike_model instance to be 
 546      the only sub-features (supplied as a dict with keys 'is_spike_data' 
 547      and 'is_spike_model'). 
 548      """ 


549 -    def _local_init(self):


550          assert len(self.subfeatures) == 2 
 551          assert remain(self.subfeatures.keys(), 
 552                        ['is_spike_data', 'is_spike_model']) == []

 553   


554 -    def postprocess_ref_traj(self):


555          # get precise spike times and record in self.results.ref_spike_times 
 556          self.pars.ref_spike_times, self.pars.ref_spike_vals = \ 
 557                self._eval(self.ref_traj, self.super_pars.ref_burst_est, 
 558                                   self.subfeatures['is_spike_data'])

 559   


560 -    def evaluate(self, target):


561          self.results.spike_times, self.results.spike_vals = \ 
 562                 self._eval(target.test_traj, self.super_results.burst_est, 
 563                                       self.subfeatures['is_spike_model']) 
 564          # satisfied if all spikes determined correctly 
 565          return len(self.results.spike_times) == \ 
 566                 len(self.super_results.burst_est.spike_ixs)

 567   
 568   


569 -    def _eval(self, traj, burst_est, is_spike):


570          # isn't the next line redundant? 
 571          is_spike.super_pars = copy.copy(self.pars) 
 572          spike_times = [] 
 573          spike_vals = [] 
 574          satisfied = True 
 575          for spike_num, spike_ix in enumerate(burst_est.spike_ixs): 
 576              if self.pars.verbose_level > 0: 
 577                  print "\n Starting spike", spike_num+1 
 578              is_spike.super_pars.burst_coord = self.super_pars.burst_coord 
 579              # step back 10% of estimated period 
 580              try: 
 581                  is_spike.pars.width_tol = burst_est.ISIs[spike_num]*.9 
 582              except IndexError: 
 583                  # one fewer ISI than spike, so just assume last one is about 
 584                  # the same 
 585                  is_spike.pars.width_tol = burst_est.ISIs[spike_num-1]*.9 
 586              is_spike.pars.tlo = burst_est.t[spike_ix] - \ 
 587                      is_spike.pars.width_tol / 2. 
 588              if self.pars.verbose_level > 0: 
 589                  print "new tlo =", is_spike.pars.tlo 
 590              # would prefer to work this out self-consistently... 
 591              #is_spike.pars.fit_width_max = ? 
 592              new_sat = is_spike(traj) 
 593              satisfied = satisfied and new_sat 
 594              # make recorded spike time in global time coordinates 
 595              if new_sat: 
 596                  spike_times.append(is_spike.results.spike_time) 
 597                  spike_vals.append(is_spike.results.spike_val) 
 598              if self.pars.verbose_level > 0: 
 599                  print "Spike times:", spike_times 
 600          return spike_times, spike_vals

 601   
 602   


603 -class get_burst_peak_env(qt_feature_leaf):


604      """Requires tol and num_samples parameters. 
 605      """ 


606 -    def _local_init(self):


607          self.metric = metric_L2() 
 608          self.metric_len = self.pars.num_samples

 609   


610 -    def postprocess_ref_traj(self):


611          # should really use quadratic fit to get un-biased peaks 
 612          peak_vals = self.super_pars.ref_spike_vals 
 613          peak_t = self.super_pars.ref_spike_times 
 614          self.ref_traj = numeric_to_traj([peak_vals], 'peak_envelope', 
 615                                          self.super_pars.burst_coord, peak_t, 
 616                                          self.super_pars.ref_burst_pts_resampled.indepvarname, 
 617                                          discrete=False) 
 618          ref_env_ts = npy.linspace(peak_t[0], peak_t[-1], 
 619                                              self.pars.num_samples) 
 620          self.pars.ref_peak_vals = self.ref_traj(ref_env_ts, 
 621                                     self.super_pars.burst_coord)[0]

 622   


623 -    def evaluate(self, target):


624          # ignore target 
 625          dc_offset = self.super_results.baseline_V 
 626          # min and max events in model mean that these are recorded 
 627          # accurately in the pointsets already 
 628          peak_vals = self.super_results.spike_vals - dc_offset 
 629          peak_t = self.super_results.spike_times 
 630          self.results.burst_peak_env = numeric_to_traj([peak_vals], 
 631                                                        'peak_envelope', 
 632                                          self.super_pars.burst_coord, peak_t, 
 633                                  self.super_pars.burst_pts_resampled.indepvarname, 
 634                                  discrete=False) 
 635  #        burst_est = self.super_results.burst_est 
 636  #        call_args = {} 
 637  #        try: 
 638  #            call_args['noise_floor'] = is_spike.pars.noise_tol 
 639  #        except AttributeError: 
 640  #            pass 
 641  #        try: 
 642  #            call_args['depvar'] = self.super_pars.burst_coord 
 643  #        except AttributeError: 
 644  #            pass 
 645  #        try: 
 646  #            call_args['tol'] = 1.1*burst_est.std_ISI/burst_est.mean_ISI 
 647  #        except AttributeError: 
 648  #            pass 
 649  #        call_args['make_traj'] = False 
 650  #        call_args['spest'] = burst_est 
 651  #        env = spike_envelope(burst_est.pts, burst_est.mean_ISI, 
 652  #                             **call_args) 
 653          test_env_ts = npy.linspace(peak_t[0], peak_t[-1], self.pars.num_samples) 
 654          return self.metric(self.results.burst_peak_env(test_env_ts, 
 655                                                     self.super_pars.burst_coord), 
 656                             self.super_pars.ref_peak_vals) < self.pars.tol

 657   
 658   


659 -class get_burst_trough_env(qt_feature_leaf):


660      """Requires tol and num_samples parameters. 
 661      """ 


662 -    def _local_init(self):


663          self.metric = metric_L2() 
 664          self.metric_len = self.pars.num_samples

 665   


666 -    def postprocess_ref_traj(self):


667          burst_pts = self.super_pars.ref_burst_pts_resampled 
 668          burst_est = self.super_pars.ref_burst_est 
 669          vals = burst_pts[self.super_pars.burst_coord] 
 670          inter_spike_ixs = [(burst_est.spike_ixs[i-1], 
 671                              burst_est.spike_ixs[i]) \ 
 672                             for i in xrange(1, len(burst_est.spike_ixs))] 
 673          # should really use quadratic fit to get an un-biased minimum 
 674          trough_ixs = [npy.argmin(vals[ix_lo:ix_hi])+ix_lo for ix_lo, ix_hi in \ 
 675                        inter_spike_ixs] 
 676          trough_vals = [vals[i] for i in trough_ixs] 
 677          trough_t = [burst_pts.indepvararray[i] for i in trough_ixs] 
 678          self.ref_traj = numeric_to_traj([trough_vals], 'trough_envelope', 
 679                                          self.super_pars.burst_coord, trough_t, 
 680                                          burst_pts.indepvarname, discrete=False) 
 681          ref_env_ts = npy.linspace(trough_t[0], trough_t[-1], 
 682                                              self.pars.num_samples) 
 683          self.pars.ref_trough_vals = self.ref_traj(ref_env_ts, 
 684                                    self.super_pars.burst_coord)

 685   


686 -    def evaluate(self, target):


687          # ignore target 
 688          dc_offset = self.super_results.baseline_V 
 689          burst_pts = self.super_pars.burst_coord_pts 
 690          burst_est = self.super_results.burst_est 
 691          vals = burst_pts[self.super_pars.burst_coord] 
 692          ts = self.super_results.spike_times 
 693          spike_ixs = [] 
 694          for t in ts: 
 695              tix = burst_pts.find(t, end=0) 
 696              spike_ixs.append(tix) 
 697          inter_spike_ixs = [(spike_ixs[i-1], 
 698                              spike_ixs[i]) \ 
 699                             for i in xrange(1, len(ts))] 
 700          # min and max events in model mean that these are recorded 
 701          # accurately in the pointsets already 
 702          trough_ixs = [npy.argmin(vals[ix_lo:ix_hi])+ix_lo for ix_lo, ix_hi in \ 
 703                        inter_spike_ixs] 
 704          trough_vals = [vals[i] - dc_offset for i in trough_ixs] 
 705          # use self.pars.trough_t for isi mid-point times 
 706          trough_t = [burst_pts.indepvararray[i] for i in trough_ixs] 
 707          self.results.burst_trough_env = numeric_to_traj([trough_vals], 
 708                                                          'trough_envelope', 
 709                                          self.super_pars.burst_coord, 
 710                                          trough_t, 
 711                                          burst_pts.indepvarname, discrete=False) 
 712          test_env_ts = npy.linspace(trough_t[0], trough_t[-1], 
 713                                     self.pars.num_samples) 
 714          self.results.trough_t = trough_t 
 715          return self.metric(self.results.burst_trough_env(test_env_ts, 
 716                                                  self.super_pars.burst_coord), 
 717                             self.super_pars.ref_trough_vals) < self.pars.tol

 718   
 719   


720 -class get_burst_isi_env(qt_feature_leaf):


721      """Requires tol and num_samples parameters. 
 722      """ 


723 -    def _local_init(self):


724          self.metric = metric_L2() 
 725          self.metric_len = self.pars.num_samples

 726   


727 -    def postprocess_ref_traj(self):


728          burst_pts = self.super_pars.ref_burst_pts_resampled 
 729          ts = burst_pts.indepvararray 
 730          burst_est = self.super_pars.ref_burst_est 
 731          # find approximate (integer) mid-point index between spikes 
 732          mid_isi_ixs = [int(0.5*(burst_est.spike_ixs[i-1]+burst_est.spike_ixs[i])) \ 
 733                             for i in xrange(1, len(burst_est.spike_ixs))] 
 734          isi_t = [ts[i] for i in mid_isi_ixs] 
 735          isi_vals = [ts[burst_est.spike_ixs[i]]-ts[burst_est.spike_ixs[i-1]] for \ 
 736                      i in xrange(1, len(burst_est.spike_ixs))] 
 737          self.ref_traj = numeric_to_traj([isi_vals], 'isi_envelope', 
 738                                          self.super_pars.burst_coord, isi_t, 
 739                                          burst_pts.indepvarname, discrete=False) 
 740          ref_env_ts = npy.linspace(isi_t[0], isi_t[-1], 
 741                                              self.pars.num_samples) 
 742          self.pars.ref_isis = self.ref_traj(ref_env_ts, 
 743                                     self.super_pars.burst_coord)

 744   


745 -    def evaluate(self, target):


746          # ignore target 
 747          ts = self.super_results.spike_times 
 748          tname = self.super_pars.burst_coord_pts.indepvarname 
 749          isi_vals = [ts[i]-ts[i-1] for i in xrange(1, len(ts))] 
 750          self.results.burst_isi_env = numeric_to_traj([isi_vals], 
 751                                                          'isi_envelope', 
 752                                          self.super_pars.burst_coord, 
 753                                          self.super_results.trough_t, 
 754                                          tname, discrete=False) 
 755          test_env_ts = npy.linspace(self.super_results.trough_t[0], 
 756                                     self.super_results.trough_t[-1], 
 757                                     self.pars.num_samples) 
 758          return self.metric(self.results.burst_isi_env(test_env_ts, 
 759                                                     self.super_pars.burst_coord), 
 760                             self.pars.ref_isis) < self.pars.tol

 761   
 762   


763 -class get_burst_upsweep(qt_feature_leaf):


764 -    def _local_init(self):


765          self.metric = metric_L2() 
 766          self.metric_len = len(self.pars.t_offs)

 767   


768 -    def postprocess_ref_traj(self):


769          vname = self.super_pars.burst_coord 
 770          ts = [self.super_pars.ref_spike_times[0] - toff for \ 
 771                toff in self.pars.t_offs] 
 772          self.pars.ref_upsweep_V = npy.array([self.ref_traj(t, vname) for \ 
 773                                               t in ts])

 774   


775 -    def evaluate(self, target):


776          dc_offset = self.super_results.baseline_V 
 777          vname = self.super_pars.burst_coord 
 778          all_pts = self.super_pars.burst_coord_pts 
 779          vals = [] 
 780          for toff in self.pars.t_offs: 
 781              target_t = self.super_results.spike_times[0] - toff 
 782              if target_t < all_pts.indepvararray[0]: 
 783                  # out of range - return penalty 
 784                  self.metric.results = 5000*npy.ones((self.metric_len,),float) 
 785                  return False 
 786              tix = all_pts.find(target_t, end=0) 
 787              new_var = make_poly_interpolated_curve(all_pts[tix-5:tix+5], 
 788                                                     vname, target.model) 
 789              vals.append(new_var(target_t)) 
 790          self.results.upsweep_V = npy.array(vals) - dc_offset 
 791          return self.metric(self.results.upsweep_V, \ 
 792                                 self.pars.ref_upsweep_V) < self.pars.tol

 793   
 794   


795 -class get_burst_downsweep(qt_feature_leaf):


796 -    def _local_init(self):


797          self.metric = metric_L2() 
 798          self.metric_len = len(self.pars.t_offs)

 799   


800 -    def postprocess_ref_traj(self):


801          vname = self.super_pars.burst_coord 
 802          ts = [self.super_pars.ref_spike_times[-1] + toff for \ 
 803                toff in self.pars.t_offs] 
 804          self.pars.ref_downsweep_V = npy.array([self.ref_traj(t, vname) for \ 
 805                                                 t in ts])

 806   


807 -    def evaluate(self, target):


808          dc_offset = self.super_results.baseline_V 
 809          vname = self.super_pars.burst_coord 
 810          all_pts = self.super_pars.burst_coord_pts 
 811          vals = [] 
 812          for toff in self.pars.t_offs: 
 813              target_t = self.super_results.spike_times[-1] + toff 
 814              if target_t > all_pts.indepvararray[-1]: 
 815                  # out of range - return penalty 
 816                  self.metric.results = 5000*npy.ones((self.metric_len,),float) 
 817                  return False 
 818              tix = all_pts.find(target_t, end=0) 
 819              new_var = make_poly_interpolated_curve(all_pts[tix-5:tix+5], 
 820                                                     vname, target.model) 
 821              vals.append(new_var(target_t)) 
 822          self.results.downsweep_V = npy.array(vals) - dc_offset 
 823          return self.metric(self.results.downsweep_V, 
 824                                 self.pars.ref_downsweep_V) < self.pars.tol

 825   


826 -class get_burst_num_spikes(qt_feature_leaf):


827 -    def _local_init(self):


828          self.metric = metric_float() 
 829          self.metric_len = 1

 830   


831 -    def evaluate(self, target):


832          return self.metric(npy.array(len(self.super_results.spike_times)), 
 833                             npy.array(len(self.super_pars.ref_spike_times))) == 0

 834   
 835   


836 -class get_burst_period_info(qt_feature_leaf):


837 -    def _local_init(self):


838          self.metric = metric_weighted_L2() 
 839          self.metric_len = 2 
 840          # strongly penalize lack of periodicity 
 841          self.metric.weights = npy.array([1., 1000.])

 842   


843 -    def evaluate(self, target):


844          return self.metric(npy.array([self.super_results.period, 
 845                                    self.super_results.period_val_error]), 
 846                             npy.array([self.super_pars.ref_period, 
 847                                    0.])) \ 
 848                 < self.pars.tol

 849   
 850  # -------------------------------------------- 
 851   
 852   


853 -class spike_metric(metric):


854      """Measures the distance between spike time and height, 
 855      using an inherent weighting of height suited to neural voltage 
 856      signals (0.05 of time distance).""" 


857 -    def __call__(self, sp1, sp2):


858          # weight 'v' component down b/c 't' values are on a different scale 
 859          self.results = npy.array(sp1-sp2).flatten()*npy.array([1,0.05]) 
 860          return npy.linalg.norm(self.results)

 861   


862 -class spike_feature(qt_feature_node):


863      """pars keys: tol""" 


864 -    def _local_init(self):


865          self.metric_len = 2 
 866          self.metric = spike_metric()

 867   


868 -    def evaluate(self, target):


869          # traj will be a post-processed v trajectory -> 
 870          #   spike time and height values 
 871          return self.metric(target.test_traj.sample(), self.ref_traj.sample()) \ 
 872                         < self.pars.tol

 873   
 874   


875 -class geom_feature(qt_feature_leaf):


876      """Measures the residual between two 1D parameterized geometric 
 877      curves (given as Trajectory objects). 
 878      """ 


879 -    def _local_init(self):


880          self.metric = metric_L2() 
 881          self.metric_len = len(self.pars.tmesh)

 882   


883 -    def evaluate(self, target):


884          # resample ref_traj to the tmesh we want 
 885          return self.metric(target.test_traj(self.pars.tmesh, 
 886                                           coords=[self.pars.depvar]), 
 887                             self.ref_traj(self.pars.tmesh, 
 888                                           coords=[self.pars.depvar])) < self.pars.tol

 889   
 890  # ------------------------------------------------------------------ 
 891   


892 -class estimate_spiking(object):


893      """Estimate pattern of spiking in tonic or burst patterns.""" 
 894   


895 -    def __init__(self, x, t, filt_coeffs, sense='up'):


896          """Pass only 1D pointset. 
 897          If spikes are in the "positive" direction of the variable, 
 898          use sense='up', else use 'down'.""" 
 899          self.sense = sense 
 900          self.b, self.a = filt_coeffs 
 901          x_filt = filtfilt(self.b, self.a, x) 
 902          self.x_just_filt = x_filt 
 903          self.t = t 
 904          max_x = max(x_filt) 
 905          # retain only values larger than 10% of max to estimate burst 
 906          # envelope 
 907          x_filt_mask = npy.asarray(x_filt>(0.1*max_x),int) 
 908          burst_off_ix = len(t) - npy.argmax(x_filt_mask[::-1]) 
 909          burst_on_ix = npy.argmax(x_filt_mask) 
 910          self.burst_on = (burst_on_ix, t[burst_on_ix]) 
 911          self.burst_off = (burst_off_ix, t[burst_off_ix]) 
 912          self.burst_duration = t[burst_off_ix] - t[burst_on_ix] 
 913          # retain only values larger than 25% of max for actual spikes 
 914          x_filt_th = npy.asarray(x_filt>(0.25*max_x),int)*x_filt 
 915          # find each spike by group of positive values 
 916          # eliminating each afterwards (separated by zeros) 
 917          spike_ixs = [] 
 918          done = False 
 919          n = 0  # for safety 
 920          while not done: 
 921              # find next group centre and eliminate it 
 922              x_filt_th = self.eliminate_group(x_filt_th, spike_ixs) 
 923              n += 1 
 924              # no groups left to eliminate? 
 925              done = max(x_filt_th) == 0 or n > 100 
 926          spike_ixs.sort() 
 927          self.spike_ixs = spike_ixs 
 928          self.spike_ts = t[spike_ixs] 
 929          self.ISIs = [self.spike_ts[i]-self.spike_ts[i-1] for \ 
 930                       i in xrange(1, len(spike_ixs))] 
 931          self.mean_ISI = npy.mean(self.ISIs) 
 932          self.std_ISI = npy.std(self.ISIs) 
 933          self.num_spikes = len(spike_ixs)

 934   


935 -    def eliminate_group(self, xf, spike_ixs):


936          centre_ix = npy.argmax(xf) 
 937  #        print "Current spike_ixs", spike_ixs 
 938  #        print "eliminating group at t = ", self.t[centre_ix] 
 939          # forward half-group 
 940          end_ix = npy.argmin(xf[centre_ix:])+centre_ix 
 941          # backward half-group 
 942          start_ix = centre_ix-npy.argmin(xf[:centre_ix+1][::-1]) 
 943          # nullify values in range! 
 944          xf[start_ix:end_ix]=0 
 945  #        print start_ix, end_ix, xf[start_ix:end_ix] 
 946          if self.sense == 'up': 
 947              # x will be rising to peak, so track forwards until 
 948              # xfilt makes zero crossing and becomes negative 
 949              new = centre_ix+npy.argmax(self.x_just_filt[centre_ix:]<0) 
 950              if new not in spike_ixs: 
 951                  spike_ixs.append(new) 
 952          else: 
 953              # track backwards 
 954              new = centre_ix-npy.argmin(self.x_just_filt[:centre_ix+1]>0) 
 955              if new not in spike_ixs: 
 956                  spike_ixs.append(new) 
 957          return xf

 958   
 959   


960 -class spike_envelope(object):


961      """Find an amplitude envelope over a smooth 1D signal that features 
 962      roughly periodic spikes. Input is a 1D parameterized pointset 
 963      and the approximate period. An optional input is the tolerance (fraction) 
 964      for finding spikes around the period (measuring uncertainty in the 
 965      period) -- default 0.2 (20% of the period). 
 966   
 967      Optional start_t sets where to orient the search in the independent 
 968      variable -- default None (start at the highest point of the signal). 
 969      It *must* be a value that is present in the independent variable 
 970      array of the given points argument. 
 971   
 972      Optional noise_floor sets minimum signal amplitude considered to 
 973      be a peak (default 0 means non-noisy data assumed). 
 974   
 975      Outside of spike times +/- tol, envelope curve will be defined as 
 976      amplitude zero. 
 977   
 978      adjust_rate is a fraction < 1 specifying the %age change of spike 
 979      search interval (a.k.a. 'period'). default 0.1. 
 980   
 981      make_traj option can be used to avoid costly creation of a Trajectory 
 982      object representing the envelope curve, if unneeded (default True). 
 983   
 984      When less is known in advance about the regularity or other properties 
 985      of the spikes, pre-process using estimate_spiking() and pass the 
 986      result as the optional argument spest. 
 987      """ 


988 -    def __init__(self, pts, per, tol=0.2, start_t=None, 
 989                   noise_floor=0, depvar=None, adjust_rate=0.1, 
 990                   make_traj=True, spest=None):


991          try: 
 992              self.tvals = pts.indepvararray 
 993          except: 
 994              raise TypeError("Parameterized pointset required") 
 995          self.pts = pts  # store this to take advantage of index search 
 996          if depvar is None: 
 997              assert pts.dimension == 1 
 998              depvar = pts.coordnames[0] 
 999              self.vals = pts[depvar] 
1000          else: 
1001              try: 
1002                  self.vals = pts[depvar] 
1003              except PyDSTool_KeyError: 
1004                  raise ValueError("Invalid dependent variable name") 
1005          self.numpoints = len(self.vals) 
1006          assert self.numpoints > 1 
1007          self.per = per 
1008          self.noise_floor = noise_floor 
1009          assert tol < 1 and tol > 0 
1010          self.tol = tol 
1011          # assume that the maximum is a spike, so is a reliable 
1012          # phase reference 
1013          if start_t is None: 
1014              self.start_ix = npy.argmax(self.vals) 
1015              self.start_t = self.tvals[self.start_ix] 
1016          else: 
1017              assert start_t in self.tvals 
1018              self.start_t = start_t 
1019              self.start_ix = pts.find(start_t) 
1020          assert adjust_rate > 0 and adjust_rate < 1 
1021          adjust_rate_up = 1+adjust_rate 
1022          adjust_rate_down = 1-adjust_rate 
1023          spike_ixs_lo = [] 
1024          spike_ixs_hi = [] 
1025          start_t = self.start_t 
1026          per = self.per 
1027          tol = self.tol 
1028          done_dir = False 
1029          while not done_dir: 
1030  #            print "\n======================\nDir +1" 
1031              res = self.find_spike_ixs_dir(1, per=per, start_t=start_t, 
1032                                            tol=tol) 
1033              spike_ixs_hi.extend(res['spike_ixs']) 
1034              if res['success']: 
1035                  done_dir = True 
1036              else: 
1037                  if res['problem_dir'] == 'lo': 
1038                      per = per * adjust_rate_down 
1039                  elif res['problem_dir'] == 'hi': 
1040                      per = per * adjust_rate_up 
1041                  rat = per/self.per 
1042                  if rat > 2 or rat < 0.5: 
1043                      # per is too far off, must be no more spikes 
1044                      done_dir = True 
1045                      continue 
1046  #                print "failed:", res['problem_dir'], res['restart_t'] 
1047                  start_t = res['restart_t'] 
1048                  #tol *= 1.2 
1049          start_t = self.start_t 
1050          per = self.per 
1051          tol = self.tol 
1052          done_dir = False 
1053          while not done_dir: 
1054  #            print "\n======================\nDir -1" 
1055              res = self.find_spike_ixs_dir(-1, per=per, start_t=start_t, 
1056                                            tol=tol) 
1057              spike_ixs_lo.extend(res['spike_ixs']) 
1058              if res['success']: 
1059                  done_dir = True 
1060              else: 
1061                  if res['problem_dir'] == 'lo': 
1062                      per = per * adjust_rate_up 
1063                  elif res['problem_dir'] == 'hi': 
1064                      per = per * adjust_rate_down 
1065                  rat = per/self.per 
1066                  if rat > 2 or rat < 0.5: 
1067                      # per is too far off, must be no more spikes 
1068                      done_dir = True 
1069                      continue 
1070  #                print "failed:", res['problem_dir'], res['restart_t'] 
1071                  start_t = res['restart_t'] 
1072                  #tol *= 1.2 
1073          spike_ixs_lo.sort() 
1074          spike_ixs_hi.sort() 
1075          ts = self.pts.indepvararray 
1076          self.spike_ixs = npy.array(spike_ixs_lo+spike_ixs_hi) 
1077          self.spike_vals = npy.array([self.vals[i] for i in self.spike_ixs]) 
1078          nearest_per_ix_lo = self.pts.find(ts[self.spike_ixs[0]]-per*tol, end=1) 
1079          nearest_per_ix_hi = self.pts.find(ts[self.spike_ixs[-1]]+per*tol, end=0) 
1080          # fill in rest of curve (outside of +/- period tolerance) with zeros 
1081          if spike_ixs_lo == [] or spike_ixs_lo[0]==0: 
1082              # spike right at t=0 
1083              prepend_v = [] 
1084              prepend_t = [] 
1085          elif nearest_per_ix_lo==0: 
1086              # -per*tol reaches to t=0 
1087              prepend_v = [self.spike_vals[0]] 
1088              prepend_t = [ts[0]] 
1089          else: 
1090              # add zeros up to -per*tol of first spike 
1091              prepend_v = [0,0,self.spike_vals[0]] 
1092              prepend_t = [ts[0], 
1093                           ts[nearest_per_ix_lo-1], 
1094                           ts[nearest_per_ix_lo]] 
1095          if spike_ixs_hi[-1]==self.numpoints-1: 
1096              postpend_v = [] 
1097              postpend_t = [] 
1098          elif nearest_per_ix_hi==self.numpoints-1: 
1099              postpend_v = [self.spike_vals[-1]] 
1100              postpend_t = [ts[-1]] 
1101          else: 
1102              postpend_v = [self.spike_vals[-1],0,0] 
1103              postpend_t = [ts[nearest_per_ix_hi], 
1104                            ts[nearest_per_ix_hi+1], 
1105                            ts[-1]] 
1106          curve_vals = npy.array(prepend_v+[self.vals[i] for i in \ 
1107                                          self.spike_ixs]+postpend_v) 
1108          curve_t = prepend_t + list(ts[self.spike_ixs]) \ 
1109                     + postpend_t 
1110          #zeros_ixs_lo = xrange(0,spike_ixs_lo[0]) 
1111          #zeros_ixs_hi = xrange(spike_ixs_hi[-1],self.numpoints) 
1112          if make_traj: 
1113              self.envelope = numeric_to_traj([curve_vals], 'envelope', 
1114                                          depvar, curve_t, 
1115                                          pts.indepvarname, discrete=False)

1116   


1117 -    def find_spike_ixs_dir(self, dir=1, per=None, start_t=None, 
1118                             tol=None):


1119          """Use dir=-1 for backwards direction""" 
1120          if start_t is None: 
1121              t = self.start_t 
1122          else: 
1123              t = start_t 
1124          if per is None: 
1125              per = self.per 
1126          if tol is None: 
1127              tol = self.tol 
1128          assert dir in [-1,1] 
1129          if dir == 1: 
1130              # only include starting index once! 
1131              if t == self.start_t: 
1132                  spike_ixs = [self.start_ix] 
1133              else: 
1134                  spike_ixs = [] 
1135          else: 
1136              spike_ixs = [] 
1137   
1138          res = {'success': False, 'problem_dir': '', 'spike_ixs': [], 
1139                 'restart_t': None} 
1140          done = False 
1141          hit_end = False 
1142          while not done and not hit_end: 
1143              t += dir * per 
1144              t_lo = t - per*tol 
1145              t_hi = t + per*tol 
1146  #            print "\n******************* find:" 
1147  #            print "Search from t=", t, "existing spikes=", spike_ixs 
1148  #            print "per= ", per, "t_lo=", t_lo, "t_hi=", t_hi 
1149              lo_ix = self.pts.find(t_lo, end=0) 
1150              if lo_ix == -1: 
1151                  # hit end! 
1152                  lo_ix = 0 
1153              hi_ix = self.pts.find(t_hi, end=1) 
1154              # find() will not return vals > numpoints or < 0 
1155              hit_end = lo_ix == 0 or hi_ix == self.numpoints 
1156              if lo_ix == hi_ix: 
1157                  done = True 
1158                  continue 
1159              else: 
1160                  max_ix = npy.argmax(self.vals[lo_ix:hi_ix]) 
1161              # now ensure that time window was large enough to capture a true 
1162              # extremum, and not just an endpoint extremum 
1163              room_lo = lo_ix 
1164              room_hi = self.numpoints-hi_ix 
1165              look_lo = min((room_lo, 5)) 
1166              look_hi = min((room_hi, 5)) 
1167              if look_lo > 0 and max(self.vals[lo_ix-look_lo:lo_ix]) > \ 
1168                 self.vals[lo_ix+max_ix]: 
1169                  # then wasn't a true max - must de/increase per 
1170                  # depending on current dir 
1171                  res['success'] = False 
1172                  res['problem_dir'] = "lo" 
1173                  if dir < 0: 
1174                      res['spike_ixs'] = spike_ixs[::-1] 
1175                  else: 
1176                      res['spike_ixs'] = spike_ixs[:] 
1177                  res['restart_t'] = t - dir*per 
1178                  return res 
1179              if look_hi > 0 and max(self.vals[hi_ix:hi_ix+look_hi]) > \ 
1180                 self.vals[lo_ix+max_ix]: 
1181                  # then wasn't a true max - must de/increase per 
1182                  # depending on current dir 
1183                  res['success'] = False 
1184                  res['problem_dir'] = "hi" 
1185                  if dir < 0: 
1186                      res['spike_ixs'] = spike_ixs[::-1] 
1187                  else: 
1188                      res['spike_ixs'] = spike_ixs[:] 
1189                  res['restart_t'] = t - dir*per 
1190                  return res 
1191              if abs(self.vals[max_ix+lo_ix]-self.vals[lo_ix]) >= self.noise_floor: 
1192                  # need equals case at endpoint when lo_ix = 0 so LHS is zero but 
1193                  # maximum is on first index 
1194                  spike_ixs.append(max_ix+lo_ix) 
1195              # else don't treat as a spike 
1196   
1197          if dir < 0: 
1198              res['spike_ixs'] = spike_ixs[::-1] 
1199          else: 
1200              res['spike_ixs'] = spike_ixs[:] 
1201          res['success'] = True 
1202          return res

1203
```

  


| Home | Trees | Indices | Help | PyDSTool |
| --- | --- | --- | --- | --- |

|  |  |
| --- | --- |
| Generated by Epydoc 3.0.1 on Fri May 4 15:24:15 2012 | http://epydoc.sourceforge.net |
